# Real-World Data on Faricimab Switching in Treatment-Refractory Neovascular Age-Related Macular Degeneration

**DOI:** 10.3390/life14020193

**Published:** 2024-01-29

**Authors:** Benjamin Ng, Hema Kolli, Naduviledeth Ajith Kumar, Matthew Azzopardi, Abison Logeswaran, Julius Buensalido, Bushra Mushtaq, Randhir Chavan, Yu Jeat Chong

**Affiliations:** 1 Birmingham and Midland Eye Centre, Dudley Road, Birmingham B18 7QH, UKn.ajithkumar@nhs.net (N.A.K.); j.buensalido1@nhs.net (J.B.);; 2 Christ Church, University of Oxford, St. Aldate’s, Oxford OX1 1DP, UK; 3 Royal Free Hospital, Pond Street, London NW3 2QG, UK; matthew.azzopardi.14@um.edu.mt; 4 Moorfields Eye Hospital, 162 City Road, London EC1V 2PD, UK; abison.logeswaran.1@city.ac.uk

**Keywords:** faricimab, neovascular age-related macular degeneration, anti-VEGF, Ang-2

## Abstract

Faricimab is a newly approved bispecific antibody for neovascular age-related macular degeneration (nAMD). Our study aims to evaluate clinical outcomes of faricimab switching in patients with treatment-refractory nAMD; determine parameters that predict these outcomes; and obtain patient subjective experience on this new injection. This is a retrospective case review with clinical and imaging data from a tertiary referral unit (Birmingham and Midland Eye Centre, UK), involving patients who were switched to faricimab between 1 January and 1 December 2023. In all, 63 eyes (54 patients) with a mean age of 79.2 ± 7.8 and mean of 41.5 ± 22.4 previous anti-VEGF injections were analysed. With a mean of 4.81 ± 1.16 faricimab injections over 6.98 ± 1.75 months, post-treatment visual acuity was logMAR 0.49 ± 0.36 and central macular thickness (CMT) was 320.3 ± 97.9 µm. After first dose, 39.1% achieved complete dryness and 89.1% had anatomical improvement. Presence of subretinal fluid was a predictor of better functional outcomes (*p* = 0.001, β = −0.182), while initial CMT predicted better anatomical outcomes (*p* = 0.001, β = 0.688). Compared to their experiences of previous anti-VEGF injections, 89% of patients reported no more discomfort and 87.0% experienced no more floaters, photopsia, or bubbles post-injection. Faricimab switching has anatomical efficacy but limited functional improvement in treatment-refractory AMD. Patient experiences of faricimab compared to previous injections were overall positive.

## 1. Introduction

Age-related macular degeneration (AMD) is a leading cause of blindness in developed countries, with an estimated global prevalence of around 200 million [1,2,3]. The neovascular subtype of late AMD (nAMD) is treatable and is characterised by abnormally permeable choroidal or macular neovascularisation (MNV) that causes exudative leakage and bleeding into the macula. This leads to subacute vision loss and potentially irreversible damage to photoreceptors and retinal pigment epithelium (RPE) cells if left untreated [4,5].

In the past, the paradigm for treating nAMD was to target culprit MNVs with photocoagulation or photodynamic therapy (PDT). Photocoagulation was the first therapy described for nAMD, but it is a destructive process that kills surrounding retinal cells. Furthermore, it merely slows down visual loss, and 59% developed recurrence [6]. In an attempt to develop retinal-sparing treatments, PDT with verteporfin (vPDT) was developed. Verteporfin tends to accumulate in vessels with an abnormally low-density lipoprotein receptor such as “classic” subretinal MNV. Following irradiation with a 689 nm wavelength laser light targeted at the lesion, localised oxidative stress and a subsequent immune response lead to non-thermal vaso-occlusive damage of the photosensitised subretinal MNVs [7]. However, vPDT had variable results. The TAP trial demonstrated that patients were less likely to develop vision loss with vPDT in comparison to the placebo for occult and classic MNVs that were no more than 5400 microns in size, over 24 months. However, there was a relative risk of 0.77 for treated patients to lose 15 letters or more after 2 years (around five treatments), and that severe acute vision loss could result after only 1 week of treatment in 2% of the patients [8]. Nevertheless, PDT still has a place nowadays in treating certain nAMD subtypes such as polypoidal choroidal vasculopathy (PCV) [9].

A pivotal landmark in the treatment of nAMD was the discovery of vascular endothelial growth factor (VEGF), one of the most important molecular mediators and an important survival factor that supports endothelial cells in nAMD. Its overexpression is implicated in the formation of aberrant new vessels in the context of structural abnormalities, as in the case of AMD [10,11]. The effectiveness of VEGF suppression in pre-clinical nAMD models eventually led to a major paradigm shift with the development of intravitreal anti-VEGFs. The first molecule that was used was pegaptanib, a pegylated anti-VEGF aptamer that competitively binds the VEGF-165 isoform. In the VISION trial, it showed superior efficacy compared to sham injections for all types or sizes of neovascular membranes (NVM) [12]. However, this aptamer was limited by its durability, and was subsequently superseded by monoclonal antibodies such as ranibizumab, a recombinant humanised IgG1 Fab fragment against VEGF-A. The MARINA trial showed that ranibizumab was able to maintain a gain in visual acuity for 24 months compared to sham controls in minimally classic and occult MNV [13]. Meanwhile, the ANCHOR trial demonstrated a greater visual acuity benefit of ranibizumab in comparison to PDT in predominantly classic, subfoveal MNV [14].

Over the following decade, structural variants of anti-VEGF were developed and showed similar efficacy to ranibizumab, including bevacizumab, the complete IgG monoclonal antibody that binds all VEGF isoforms (demonstrated in the comparison of age-related macular degeneration treatment trials, CATT) and aflibercept, the decoy receptor of VEGF-A (demonstrated in the VIEW1/2 trials) [15,16,17]. The latest monospecific anti-VEGF variant, a single-chain variable fragment (scFv) antibody called brolucizumab, had initially shown promise due to its superior morphological outcomes in nAMD. However, its use has been limited due to safety concerns, as up to 30% of patients developed retinal vasculitis and/or retinal vascular occlusion [18,19].

Another revolution in ophthalmology that aided the widespread adoption of anti-VEGF treatments was the development of optical coherence tomography (OCT) around the late 90s [20]. The ease and non-invasiveness of this imaging modality meant that patients could be monitored easily, replacing routine use of fundus fluorescein angiography. Naturally, OCT has been utilised in major landmark trials on anti-VEGF for monitoring of structural outcomes. This has led to the concept of treatment intervals which now holds major importance as a treatment outcome for all intravitreal anti-VEGF therapy [13,14,17].

Until recently, anti-VEGFs remained the only available pharmacological treatment for nAMD [13,14,17,18], with the maximum posological dosing interval at 12 weeks despite the myriad of structural variants. This has now changed with the newest addition to the therapeutic armamentarium faricimab, a bispecific antibody that not only targets VEGF-A, but also angiopoietin-2 (Ang-2), which has shown extended durability beyond the 12 week interval [21,22,23]. The Ang-2/Tie2 pathway is in opposing balance with the VEGF pathway, where the inhibition of the former and activation of the latter stimulates the growth of new vessels. Ang-2 is normally present in low levels, but in pathological states a high level of Ang-2 competitively inhibits Tie2 receptors on endothelial cells by preventing the constitutively expressed Ang-1 from binding. This leads to reduced vessel maturation and stability [23,24,25,26]. Faricimab can bind both free VEGF-A and Ang-2 to reduce this angiogenic switch.

The translational efficacy of this novel drug has been demonstrated in two recent, identically designed, double-masked, and randomised controlled trials (TENAYA and LUCERNE). Faricimab was found to be non-inferior to aflibercept at primary endpoint visits (week 40, 44, and 48) in terms of best corrected visual acuity (BCVA). This was supported by improvements in anatomical outcomes (central subfield thickness) on spectral domain OCT (SD-OCT) in comparison to initial scans. More importantly, both trials highlighted the main benefit of faricimab, which is the ability to safely extend injection intervals longer than other existing monospecific anti-VEGFs. Around 45% of patients were maintained on inter-treatment intervals of 16 weeks, and around 80% reached intervals of 12 weeks or more [17]. This durability has significant impact on the health burden of patients by reducing hospital visits and potential side effects associated with each intravitreal injection.

However, the results of both randomised controlled trials do not accurately represent real-world practice, as only treatment-naïve, new nAMD patients were recruited. In practice, many patients are already on anti-VEGF treatments with treatment-resistant disease activity. These patients likely have a significant treatment burden and are in an opportune position to be switched to faricimab to reduce injection frequency. Thus far, early real-world data in the literature has shown mixed results due to short follow-ups, a heterogeneous patient population, variable previous anti-VEGF usage, and different faricimab treatment protocols [27,28,29,30,31,32]. However, there is an additional lack of real-world data on switching to this drug in patients with nAMD disease activity resistant to multiple and extensive intravitreal injections with other anti-VEGFs. In particular, it is of great interest to understand if faricimab can reduce intraretinal and subretinal fluid in treatment-resistant diseases, which is a key determination for interval extension. Finally, as patients with refractory nAMD have had multiple other anti-VEGF injections, it is also important to compare whether faricimab causes similar levels (or better/worse) of discomfort or post-procedure side effects in comparison to their previous treatments.

Our study, therefore, aims to evaluate the anatomical and functional outcomes of faricimab switching in our patient cohort and to determine parameters that predict these outcomes. Our secondary objective is to determine the perceived experience of patients receiving this medication.

## 2. Materials and Methods

### 2.1. Patient Demographics

A retrospective cohort study was performed using clinical data collected from a tertiary referral unit (Birmingham and Midland Eye Centre), between 1 January and 1 December 2023. All nAMD patients who had had multiple previous anti-VEGF treatments and had been switched to faricimab were identified using the electronic patient record system, Medisoft (Medisoft Limited, Leeds, UK). Clinical demographics including age, gender, ethnicity, lens status, ocular co-morbidities, number and types of previous intravitreal injections, and previous treatment intervals were collected.

### 2.2. Disease Definition, Inclusion and Exclusion Criteria

There is a lack of consensus on definitions of the suboptimal response to anti-VEGFs in the literature [33,34]. Strictly speaking, “non-response” describes functional response whereby there is a decline in BCVA, whereas the terminology “poor response” refers to no change in BCVA or a change of no more than four letters loss.

For morphological responses, the terms suboptimal or poor responses are often used interchangeably to describe deterioration of lesion morphology despite being on optimum intervals, but include reduction of subretinal fluid (SRF), intraretinal fluid (IRF), or central macular thickness (CMT) from 25 to 75% of baseline values. Fung et al. additionally described recalcitrant MNV as persistent IRF or SRF on SD-OCT at <30 days after the last of six monthly anti-VEGF treatments, whilst another group defined recalcitrant disease as fluid activity at 6 months of monthly anti-VEGF injections [35,36]. The SAVE trial defined recalcitrant disease as persistent IRF or SRF on SD-OCT and FFA in patients who had received nine injections in 12 months [35,37]. Recent real-world studies on faricimab switching defined persistent disease as patients receiving six injections in the previous 12 months or four injections in the previous 6 months with persistent fluid on SD-OCT [38,39,40]. Finally, as presence of exudation does not always correlate with functional decline, some ophthalmologists reserve the term “treatment-refractory” for patients with persistent fluid on SD-OCT but worsening vision despite being on active anti-VEGF treatment, and instead use “incomplete response” as presence of fluid on OCT, but relatively stable vision whilst on active treatment [41].

Therefore, we have included patients who have been switched to faricimab and met all three criteria: (1) poor or non-response functionally as defined above, (2) suboptimal or poor responses anatomically as defined above, and (3) received at least six injections in the last 6 months or at least four injections in last 6 months with persistent fluid on SD-OCT assessment [23,34].

Patients with less than two doses of faricimab or significant ocular co-morbidities were excluded from the analysis.

### 2.3. Faricimab Treatment Protocol

In our cohort, patients were switched to faricimab in three different ways. Most patients had four loading (monthly) doses of faricimab (similar to the TENAYA and LUCRENE trials) and, thereafter, were placed on a treat-and-extend regimen. Some patients had an accelerated loading, i.e., less than four faricimab before the treat-and-extend regimen commences. Finally, others were placed on “interval-matching”, where they do not receive any monthly loading doses and were immediately matched to the previous interval (longer than monthly) of anti-VEGF.

For the treat-and-extend protocol after the last of the loading doses (initiation phase), patients were extended by 4 weeks if there were improvements based on SD-OCT parameters. If there was no morphological improvement or deterioration, the patients were continued on monthly treatment until next review. All patients receive SD-OCT imaging before a pre-determined injection, with the images reviewed either during clinic, or virtually in between the patients’ visits to determine treatment interval. After an initial 4 week extension, clinicians would decide on a decrease or increase in intervals by 2 or 4 weeks (up to the maximum posological interval of 16 weeks).

### 2.4. Outcomes

Functional outcomes assessed included BCVA (logMAR) before and after treatments at the patient’s last clinical visit. The anatomical outcomes analysed included CMT and type of retinal fluid (SRF or IRF) on SD-OCT (Spectralis, Heidelberg Engineering, Heidelberg, Germany) at baseline and post-treatment. Improvements in SRF/IRF and dryness upon switching after one dose of faricimab were also noted. Anatomical dryness was defined as the absence of SRF and IRF on SD-OCT images.

### 2.5. Complications and Patient-Reported Experience

Any complications arising from faricimab treatment were documented and noted if they are specific to the drug itself. Patients were also telephoned to obtain their subjective experience on faricimab compared to previous anti-VEGF injections, and were asked about their comfort during the procedure and bubbles, photopsia, or floaters post-procedure.

### 2.6. Statistical Analysis

Statistical analysis was performed using SPSS Version 24.0.0 (IBM Corp, Armonk, New York, NY, USA). The normality of data was assessed using the Shapiro–Wilk test. Non-parametric paired data was analysed using Wilcoxon Signed Rank Test. A stepwise linear regression model was used to determine which variables (from clinical demographics and baseline treatment parameters) best predicted functional and structural outcomes, with the Durbin–Watson statistic applied to detect auto-correlation.

## 3. Results

### 3.1. Patient Demographics and Treatment Summary

A total of 63 eyes (54 patients) were identified and their baseline demographics and parameters that were analysed are summarised in Table 1.

Overall, the mean age of our patient cohort was 79.2 ± 7.8, which mainly comprised Caucasians (62.9%) and females (61.1%). Of our patients, 19.5% had ocular co-morbidities, but none of them had severe ocular pathologies, e.g., other outer retinal problems or severe/end-stage glaucoma that would represent confounding factors for outcomes.

The complexity of our cohort is highlighted by our patients having had a large number of previous anti-VEGF injections (mean: 41.5) over a long time period (mean: 65.4 months). The two commonest previous anti-VEGFs used were aflibercept (87.3%) and ranibizumab (77.8%). Of our patients, 69.8% were on four-weekly anti-VEGF treatment intervals. Eighteen patients were on longer intervals mainly due to the previous treatment protocol for brolucizumab (all 14 patients who last received brolucizumab as their last anti-VEGF before switching were extended to their matched interval of 8 weeks).

The baseline BCVA was logMAR 0.47 ± 0.34 and CMT 344.0 ± 96.1 μm. The poor baseline vision, compared to other real-world studies in the literature, reflects neuroanatomical damage from treatment-refractory nAMD.

Over 6.98 ± 1.75 months, eyes received a mean of 4.81 ± 1.16 faricimab injections (range: 2–7). In all, 49/63 (77.8%) eyes had four faricimab loading doses, 11/63 (17.4%) had accelerated loading (one dose: 1, two doses: 6, three doses: 4), 2/63 (3.2%) had no loading doses (started 8 weeks apart), and 1/63 (1.6%) had been matched to the same interval (more than 4 weeks apart, without loading doses) as their previous anti-VEGF.

### 3.2. Functional and Structural Outcomes

Outcomes of faricimab switching are summarised in Table 2.

There was no statistically significant improvement in the final BCVA (Z = −0.826 *p* = 0.41). However, a significant reduction in CMT was noted with a mean final CMT (Z = −2.47, *p* = 0.014).

Compared to baseline scans, 47/63 eyes (73.4%) showed improvement of IRF/SRF on SD-OCT 4 weeks after their last faricimab injection. After switching to the first dose of faricimab, 25/63 eyes (39.1%) had complete dryness and 57/63 eyes (89.1%) had improvement in SRF/IRF.

The average clinically stable maintenance interval was extended to 5.25 ± 1.99 weeks (baseline maintenance interval: 5.24 ± 1.90 weeks with 69.8% of patients previously on four-weekly interval).

### 3.3. Predictors of Functional Outcome

From our stepwise linear regression model used to determine factors predicting final BCVA, there was no auto-correlation confirmed by the Durbin–Watson statistic (2.49). The overall regression was statistically significant (R2 = 0.87, F(7, 53) = 50.7, *p* < 0.001). This model indicates that 87% of the variance in the final visual acuity can be explained by (1) initial BCVA (*p* = 0.001, t = 16.6, β = 0.87), (2) improvement after initial faricimab injection (*p* = 0.001, t = 3.6 β = −0.182), (3) fluid change compared to baseline scan (*p* = 0.012, t = 2.6, β = 0.138), (4) presence of SRF (*p* = 0.016, t = 2.5 β = −0.182), (5) female gender (*p* = 0.026, t = 2.3 β = 0.124), and (6) age (*p* = 0.037, t = 2.14, β = 0.116).

### 3.4. Predictors of Structural Outcome

From the stepwise linear regression model used to determine factors predicting final CMT, there was no auto-correlation confirmed by the Durbin–Watson statistic (2.25). The overall regression was statistically significant (R2 = 0.46, F(1, 59) = 53.0, *p* < 0.001). This model indicates that 46% of the variance in the final CMT was determined by only one factor: the initial CMT (*p* = 0.001, t = 7.3, β = 0.688).

There were no predictors for first-dose complete dryness upon switching to faricimab.

### 3.5. Associated Complications

3/63 (4.8%) eyes had complications associated with intravitreal injections. Two patients had minor complications including a conjunctival ulcer and a corneal abrasion. These were not faricimab-specific complications. One patient had a radiologically confirmed ischaemic stroke after their third faricimab dose, and any further anti-VEGF treatment was stopped. No patients had intraocular pressure rise, intraocular inflammation, lens touch, or endophthalmitis.

### 3.6. Patient-Reported Outcomes

85.2% of our patients (46/54 patients; 46/63 eyes) responded when contacted. Compared to other previous anti-VEGF injections, 89.1% (41/46) reported no more discomfort and 87.0% (40/46) experienced no more floaters, photopsia, or bubbles post-procedure.

## 4. Discussion

Faricimab switching offers a potential avenue for treating persistent nAMD activity after other anti-VEGF options have been exhausted. Our data mirrors other early real-world studies with respect to anatomical efficacy, even in our treatment-refractory cohort (Table 1). There were no significant functional improvements, once again reflecting the chronicity of the disease, which had resulted in structural macular changes.

There are many reasons why patients have poor morphological responses to anti-VEGFs. Firstly, there could be suboptimal treatment intervals. This is not applicable to our patients, where nearly 70% of our cohort were on four-weekly injections prior to switching. Secondly, there could be changes in the microenvironment or metabolic landscape in chronically active nAMD which alters the pharmacokinetics and pharmacodynamics. This includes higher VEGF concentrations, upregulation of other growth factors, chronic inflammation, and even neutralising antibodies against anti-VEGF. This makes targeting VEGF less efficacious over time, which is a concept called tolerance [34,42,43,44]. Tolerance may be overcome by increasing dosage, reducing treatment intervals, or targeting different pathways. The latter strategy has been employed in faricimab via targeting Ang-2, and is also illustrated by studies using adjuvant steroid treatments in treatment-refractory nAMD [23,45,46]. Tachyphylaxis is another phenomenon that leads to a progressive and rapid decrease in pharmacological response after frequent, repeated administration of a drug [47]. Tachyphylaxis can be circumvented by switching to another type of anti-VEGF, which is not an option for patients who have been trialed on various types of anti-VEGFs [48,49]. Some subtypes of nAMD may be less responsive to anti-VEGF. This was highlighted in the EVEREST-II trials where PCV responded better (higher polyp closure rate, absence of disease activity, and better BCVA gain) with a dual treatment of PDT and ranibizumab versus ranibizumab monotherapy [50]. Finally, there may be some (albeit limited) genetic influence on anti-VEGF response which requires further studies [51,52,53].

Improvements and dryness upon switching after one dose of faricimab were of particular interest in refractory disease because of the implications of overcoming the problem of anti-VEGF tolerance. Almost 40% of our patient cohort experienced first-dose dryness upon switching to faricimab and nearly 75% had reduced SRF or IRF with one dose, despite having previously received prolonged courses of many anti-VEGF intravitreal injections (mean of 41.5 injections over 65.4 months, with an average of two different types). At the latest follow-up (mean of 6.98 months and 4.81 ± 1.16 faricimab injections), 89.1% of patients exhibited structural improvement compared to their baseline SD-OCT scan. There is no consensus of the definition of treatment-refractory AMD [33,34,38,39,40]. Our cohort fits most of the criteria defined by experts in the literature. To our knowledge, our cohort also exceeds the highest mean number of anti-VEGF injections before switching and disease duration amongst the real-world studies for faricimab switching [27,28,29,30,31,32,38,39,40,54]. Despite this, our anatomical outcomes were comparable to other real-world studies, all of which showed improvements in CMT, highlighting the effective drying property of faricimab.

As alluded to earlier, anti-VEGF tolerance and tachyphylaxis are important issues in treatment-resistant nAMD. Therefore, one hypothesis for the effective drying effect of faricimab may be due to its dual blockade of VEGF-A and Ang-2. Although a previous rabbit model showed that the monospecific anti-VEGF aflibercept reduces Ang-2 levels and gene expression, the Ang-2 suppression does not appear to translate into humans [55]. This is evident from data from four phase III trials with over 3000 patients that showed it has no Ang-2 effect in nAMD or diabetic macular oedema [56]. On the other hand, dual Ang-2/VEGF-A inhibition in two murine models have demonstrated reduced MNV compared to variable outcomes for either Ang-2 or VEGF-A inhibition alone. Moreover, only dual inhibition decreased the lesion leakage area. Both MNV and leakage reduction persisted for 5 weeks in the dual inhibition group. The authors suggested that these observations may be due to Ang-2 having a role in preventing vascular permeability in response to retinal ischaemia/reperfusion injury [57]. Further supporting this, aqueous humour samples taken from patients in the phase II/III faricimab nAMD trials have demonstrated suppression of median Ang-2 levels by around 80% at week 8 of post-injection, which is sustained until week 16. The VEGF-A levels were initially suppressed but returned to baseline by week 16 [58]. Furthermore, the head-to-head comparison of the dosing phase of the TENAYA and LUCERNE trials showed that faricimab had greater reduction in central subfield thickness and resolution of SRF and IRF compared to aflibercept [59].

Another explanation for the drying effect of faricimab is that it can overcome anti-VEGF tolerance through the use of a higher concentration of antibodies (6 mg for faricimab) compared to other licensed anti-VEGF formulation (e.g., 0.5 mg for ranibizumab, 2 mg for aflibercept). This has also led to an interest in administrating higher dose anti-VEGF such as 8 mg aflibercept, which has so far demonstrated durability and effectiveness in initial studies [60]. In the future, a direct comparison between high-dose aflibercept and faricimab should discern this confounding factor and evaluate if anatomical outcomes are predominantly an anti-Ang-2 effect or merely due to higher anti-VEGF dosage.

On the other hand, we found no statistically significant functional improvement. This agreed with the results of some real-world studies and may be a reflection of disease chronicity and resultant retinal structural changes in our patient population [40,54]. In real-world studies that have shown improvement in BCVA on faricimab switching, we postulated that this is due to differences in patient cohorts whereby patients were switched to faricimab before permanent structural changes to their retina occurred. This is supported by the fact that (1) a patient cohort has a much better baseline BCVA compared to ours, and/or (2) fewer anti-VEGF injections compared to our cohort [29,38]. Even so, while the change of BCVA may be statistically significant, it may not be clinically significant. For example, in the study by Leung et al., there was an improvement of only 0.06 ≈ three letters (around half a line) in a patient cohort with long-term anti-VEGF usage (a mean of 34.2 injections over 182 weeks) [29]. This may have important implications for the timing of faricimab switching; once the criteria for treatment-refractory nAMD are identified, patients may have a potential functional benefit from a faricimab switch before permanent neuroanatomical damage occurs. Further studies are needed to identify exactly when patients benefit maximally from switching from monospecific anti-VEGF to bispecific anti-VEGF/Ang2 therapy.

Our regression model revealed that the most important determinant of final VA was the initial VA. Other factors included the initial response to treatment, anatomical change in fluid levels, and to a weaker degree, age and gender. We found that the presence of SRF was predictive with improved functional outcomes. This draws a similarity to the results of the post hoc analyses from the HARBOR trial, where the presence of residual SRF at 12 and 24 months in nAMD treated with ranibizumab was associated with better BCVA compared to resolved SRF [61]. The complete resolution of this SRF usually improved or maintained (in 91% of eyes), but sometimes worsened (in 9% of eyes), final BCVA [62,63]. The explanation for the latter group is unclear, but it is postulated that residual SRF may confer a protective barrier against atrophy or contain growth factors to preserve photoreceptor function [63,64,65,66]. Both regression models found that the number of injections did not have any predictive capacity in determining the final VA or final CMT.

After a total of 303 injections in 57 patients (63 eyes), no significant intraocular complications such as endophthalmitis, inflammation, or raised intraocular pressures were experienced. Additionally, almost 90% of our patients reported that faricimab did not cause more discomfort, photopsia, or floaters compared to previous anti-VEGF injections. Taken together, faricimab appears to be a safe medication with no added significant effect on patient experience. Our follow-up period is currently too short to quantify the impact of treatment extension in relation to improving their quality of life.

Our study has a few limitations. Firstly, it is retrospective in nature with its associated biases and heterogeneity of baseline and treatment characteristics. Secondly, our study had a relatively small number of patients. Thirdly, our follow-up (mean of around 7 months) was not long enough to provide robust data on the durability of extension intervals with faricimab. This was also hindered by interval extension being at the discretion of clinicians, which is usually initially 4 weeks when fluid-free on SD-OCT after loading doses, then 2 or 4 weeks thereafter. Thus far, only one real-world study has been designed to focus on fluid-free intervals as a primary outcome, and it is unique in that it is a prospective study. Grimaldi et al. investigated treatment durability after faricimab switching in patients (n = 26 eyes) with poor response to conventional anti-VEGFs [39]. They used four loading doses of faricimab before treat-and-extend (+/−2 week interval) over a median of 30.2 weeks. They included a disease activity assessment at week 20, and all patients were given a faricimab injection then. They found that all of their patients maintained a fluid-free OCT scan, and their median maximum fluid-free interval was 6 weeks, which was statistically significant compared to traditional anti-VEGFs. Of their patients, 48% had a sustained response at the 8 week interval (q8 week). Their anatomical response appears similar to ours over the same follow-up period (7 months); furthermore, there was first-dose complete drying with the majority of their patients, a statistically significant improvement in central subfield thickness, and no statistically significant improvements in BCVA.

Our final limitation is that not all patients have had a formal fluorescein angiogram or OCT-angiogram to determine the type of MNV responsible for leakage, hence it was not possible to determine if faricimab is more effective in one particular type of MNV. The majority of our cohort was Caucasian and only around 10% were identified as Asians. This suggests that we would expect a low number of PCV patients. However, we acknowledge that 27.8% had unspecified ethnicity on electronic health records, which potentially means that we could have underestimated the actual proportion of the Asian population in our cohort. This was also a key limitation of the TENAYA and LUCERNE trials, where there were a significant lack of Asian patients recruited [23]. This is because PCV, which is less responsive to anti-VEGF treatments, accounts for up to 60% of Asian nAMD patients [9,67,68,69]. Currently, there are some real-world studies conducted in Japan which have a decent proportion of patients with PCV, but their results are limited by their heterogeneous faricimab treatment protocol and short-term follow-up [30,31].

## 5. Conclusions

In conclusion, faricimab switching has anatomical efficacy but limited functional improvement in patients who have recalcitrant nAMD activity despite long-term usage of various single-target anti-VEGFs. Our results showed that the most important determinants of functional outcome and structural outcome were the initial VA and initial CMT, respectively, and that the number of injections did not correlate with these outcomes. These results have potential implications for early switching to faricimab as soon as the treatment-refractory criteria are met, and before permanent neuroanatomical damage to the retina occurs. The improvement in SRF and IRF after switching to faricimab in patients with refractory nAMD disease activity may be partly attributed to faricimab having an additional mechanism of blocking Ang-2. This hypothesis has been supported with pre-clinical and real-world data, but further studies are required to corroborate these results, as well as to address other causes of refractory disease, including patients with PCV lesions and microenvironmental or metabolic changes with tachyphylaxis.

## Figures and Tables

**Table 1:** Baseline clinical and treatment demographics.

Parameters	Mean	Standard Deviation (Range)
Age (years)	79.2	7.8 (57–92)
Gender	Male: 21 (38.8%)	
	Female: 33 (61.1%)	
Ethnicity	White: 34 (62.9%)	
	Afro-Caribbean: 0 (0.0%)	
	Asian: 5 (9.3%)	
	Others/not specified on electronic health records: 15 (27.8%)	
Laterality	Right: 30 (47.6%)	
	Left: 33 (52.4%)	
Lens status	Phakic: 35 (55.6%)	
	Pseudophakic: 28 (44.4%)	
Eyes with ocular co-morbidities	None: 51 (80.5%) Present: 12 (19.5%)	
	Fuchs endothelial dystrophy: 1 Glaucoma: 5 High myopia: 1 Epiretinal membrane: 2 RPE tear: 1 Previously treated central serous chorioretinopathy: 2	
Number of previous anti-vascular endothelial growth factor (VEGF) injections in each eye	41.5	22.4 (6–94)
Number of types of previous anti-VEGF injections in each eye	1.87	0.75 (1–3)
Types of previous anti-VEGF injections in each eye	Aflibercept: 55 (87.3%)	-
	Ranibizumab: 49 (77.8%)	
	Brolucizumab: 14 (22.2%)	
	Bevacizumab: 0 (0.0%)	
Frequency of the previous anti-VEGF type before commencing faricimab (weeks)	5.24 mean frequency	1.92 (4–10)
	4 weeks: 44 (69.8%) 8 weeks: 18 (28.6%) 10 weeks: 1 (1.6%)	
Time from first diagnosis to first faricimab in each eye (months)	65.4	39.0 (7–169)
Baseline visual acuity (VA; logMAR)	0.47	0.34 (0.00–1.20)
SD-OCT structural features before switch to faricimab	Presence of subretinal fluid: 49 (77.8%) Presence of intraretinal fluid: 19 (30.2%) Presence of both: 5 (7.9%)	
Baseline central macular thickness (CMT; µm)	344.0	96.1 (207.0–754.0)

**Table 2:** Functional and structural outcomes after faricimab switching.

Outcomes	Baseline	Final	*p* Value
Best corrected visual acuity (BCVA; logMAR)	0.47 ± 0.34 (0.00–1.20)	0.49 ± 0.36 (0.0–1.36)	0.41
Central macular thickness (CMT; µm)	344.0 ± 96.1 (207.0–754.0)	320.3 ± 97.9 (186.0–754.0)	0.014

## Data Availability

The data presented in this study are available on reasonable request from the corresponding author. The data are not publicly available due to [patient confidentiality].
